# Successful prone positioning after recent caesarean section in severe ARDS with postpartum pulmonary haemorrhage

**DOI:** 10.1002/rcr2.673

**Published:** 2020-10-13

**Authors:** Vorakamol Phoophiboon, Thitiwat Sriprasart

**Affiliations:** ^1^ Division of Pulmonary and Critical Care Medicine, Department of Medicine, Faculty of Medicine Chulalongkorn University Bangkok Thailand; ^2^ Excellence Center for Critical Care Medicine King Chulalongkorn Memorial Hospital, Thai Red Cross Society Bangkok Thailand

**Keywords:** Caesarean section, postpartum stage, prone positioning, pulmonary haemorrhage, severe ARDS

## Abstract

A 35‐year‐old Thai women (gravida 3, para 0) at 36 weeks and five days of gestation was admitted to a delivery room due to premature rupture of membrane. She was diagnosed with *Escherichia coli* with extended‐spectrum beta‐lactamase (ESBL) chorioamnionitis and septic shock leading to signs of fetal distress. She underwent emergency caesarean section. Post‐operatively, the patient developed severe acute respiratory distress syndrome (ARDS), disseminated intravascular coagulation (DIC), massive pulmonary haemorrhage, and intra‐abdominal bleeding. Lung protective strategy and recruitment manoeuvres were applied; however, her oxygenation and haemodynamic parameters worsened. Twenty consecutive hours of prone positioning was performed as a rescue procedure to improve patient's oxygenation and allow the patient to undertake surgical re‐exploration for abdominal compartment syndrome management safely. Neither high ventilator setting nor re‐positioning was needed after the second operation.

## Graphical abstract

We report a patient with severe acute respiratory distress syndrome (ARDS) and bleeding diathesis post‐caesarean section resulting from acute chorioamnionitis with septic shock in whom prone positioning was a rescue procedure to improve oxygenation in the context of the patient's severe condition.

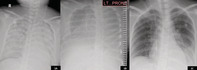

## Introduction

Prone positioning has a significant mortality benefit in acute respiratory distress syndrome (ARDS) with a ratio of arterial oxygen pressure to fractional inspired oxygen **(**PaO_2_/FiO_2_ **)** less than 150 mm Hg. Prone therapy has demonstrated several positive outcomes such as mitigating ventilation–perfusion mismatch, reducing ventilator‐induced lung injury **(**VILI**)**, promoting lung homogenous inflation, and increasing overall pulmonary compliance **[** 1]. However, there are limited evidences of its effect on patients with recent caesarean section or abdominal surgery **[** 2, 3]. We report a patient with severe ARDS and bleeding diathesis post‐caesarean section resulting from acute chorioamnionitis with septic shock in whom prone positioning was a rescue procedure to improve oxygenation in the context of the patient's severe condition.

## Case Report

**Figure 1:**
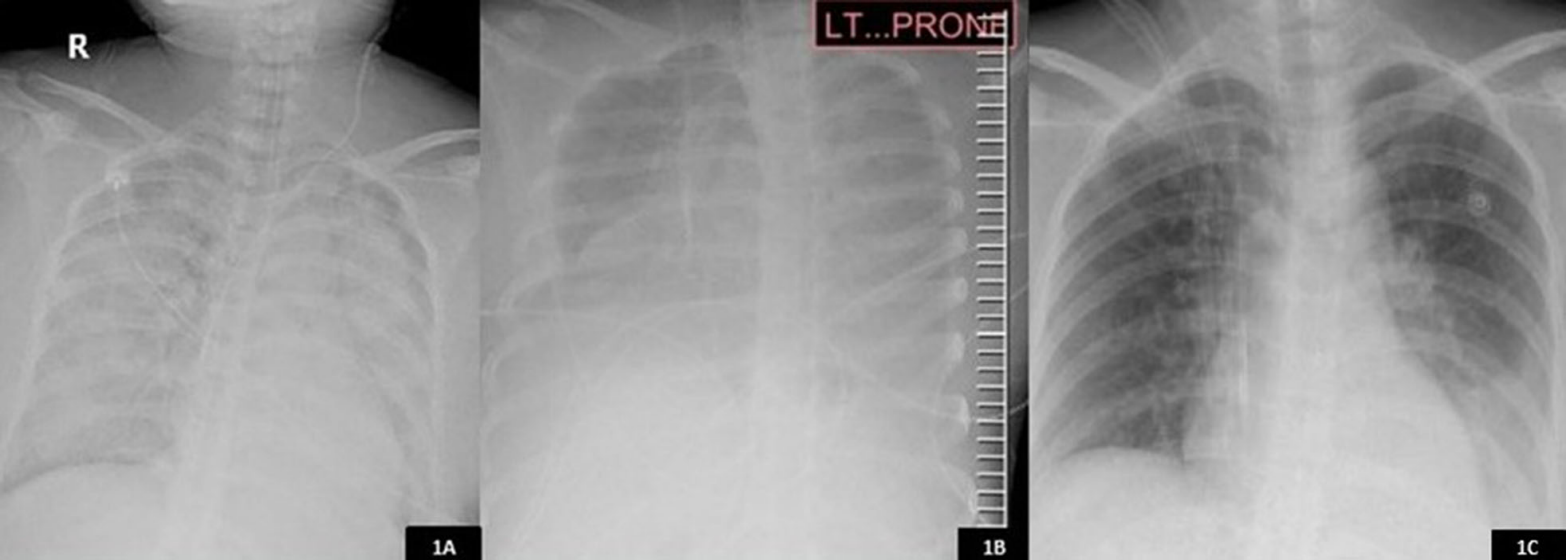
Chest radiograph at post‐caesarean section illustrated bilateral diffuse alveolar infiltration (A), at prone position (B) and at two weeks after admission (C).

Our patient was a 35‐year‐old Thai women, G3P0, 36 weeks and five days of gestation. She was admitted due to premature rupture of membrane with high‐grade fever of 39°C. Her blood pressure was 144/92 mm Hg, with heart rate of 132/min and respiratory rate of 28/min. Her initial oxygen saturation was 98% without oxygen supplement. Due to rapid progression of hypoxaemia and fetal distress, the patient underwent emergency caesarean section at midline incision under general anaesthesia. The preterm female foetus was delivered without cardiac activity. Intrauterine amniotic fluid had a foul smell. Post‐operatively, our patient developed uterine atony with multiple sites of bleeding from surgical area, endotracheal tube, and nasogastric tube. She was resuscitated with fluid, blood products, intravenous tranexamic acid, and moderate dose of norepinephrine. Intravenous meropenem was empirically given. The patient's complete blood counts revealed haemoglobin level of 6.8 g/dL decreasing from 11.2 g/dL, platelet level of 85 × 10^9^/L decreasing from 357 × 10^9^/L, and white blood cell count of 4.2 × 10^9^/L (neutrophil 92% and lymphocyte 7%). The coagulation studies revealed prothrombin time (PT) of 49 s, partial thromboplastin time (PTT) of 53 s, and international normalized ratio (INR) of 4.84. Preoperative blood culture and amniotic fluid culture revealed *Escherichia coli* with extended‐spectrum beta‐lactamase (ESBL). In terms of mechanical ventilator management, 6 mL/kg of tidal volume, high dose of sedative drugs and neuromuscular blockage agent were implemented for severe ARDS management. Although positive end‐expiratory pressure (PEEP) titration was increased up to 20 cm H_2_O (peak airway pressure of 38–40 cm H_2_O and plateau pressure of 32 cm H_2_O), the oxygen saturation was 88–90% with FiO_2_ 1 resulting in 61 mm Hg of PaO_2_/FiO_2_ and 62 mm Hg of arterial carbon dioxide pressure (PaCO_2_). There was lack of improvement in oxygenation after 6 h of high PEEP regimen; therefore, we decided to perform prone positioning with minimizing of pressure on the patient's abdomen. The chest radiograph illustrated bilateral diffuse alveolar infiltration in supine and prone positions **(**Fig. 1A, B **).** The patient's oxygenation significantly improved after 20‐h prone positioning corresponding with decreasing PEEP level and FiO_2_ to 10 cm H_2_O and 0.4, respectively **(**Table 1 **).** However, the patient's lactate, haematological profiles, and haemodynamic outcomes including peak airway pressure were worsening. At this point, we decided to turn the patient into supine position for performing diagnostic investigations. Intra‐abdominal pressure was 28 mm Hg consistent with abdominal compartment syndrome and abdominal ultrasonography revealed multiple loculated collections and blood clots (the largest collection was 4.2 × 9.1 × 13.7 cm). The patient immediately underwent decompressive laparotomy while the oxygenation was maintained in supine position. Blood clots and 2700 mL of serosanguinous fluid were removed. After abdominal decompression, the patient's haemodynamic parameters dramatically improved without continuation of prone positioning. The patient's chest radiograph on the extubation day (two weeks of admission) showed significant improvement (Fig. 1C), and she was safely discharged without morbidities.

**Table 1:** Mechanical ventilator settings and laboratory investigations.

Parameters	Post‐caesarean section (supine)	6 h After caesarean section (supine)	8 h of Prone positioning	20 h of Prone positioning before abdominal re‐exploration	6 h After abdominal re‐exploration (supine)
Mode of ventilator	VCV	VCV	VCV	VCV	VCV
Volume (mL)^*^	300	300	300	300	300
PEEP (cm H_2_O)	10	20	14	10	8
Peak airway pressure (cm H_2_O)	28	38–40	30–32	32–36	25
Plateau pressure (cm H_2_O)	22	32	N/A due to prone	N/A due to prone	18
FiO_2_	1	1	0.6	0.4	0.4
PaO_2_ (mm Hg)	64	61	77	93	135
PaCO_2_ (mm Hg)	22	62	48	31	36
PaO_2_/FiO_2_ (mm Hg)	77	61	128	232	337
Oxygen saturation (%)	90–92	88–90	94–96	98–100	100
Lactate (mmol/L)	5.6	7.3	6.5	6.9	2.1

* Patient's IBW was 50 kg.

FiO_2_, fractional inspired oxygen; IBV, ideal body weight; PaCO_2_, arterial carbon dioxide pressure; PaO_2_, arterial oxygen pressure; PEEP, positive end‐expiratory pressure; VCV, volume control ventilation.

## Discussion

Our patient presented *E. coli* ESBL chorioamnionitis with septic shock and severe ARDS which developed post‐operatively. Despite being on high PEEP titration and paralysis protocol, the oxygenation did not improve. The prone therapy has recently been recommended for severe ARDS treatment; however, the uncertainty of this procedure remains in patients with severe ARDS and either pregnancy or post‐caesarean section stage due to scarce reports [4, 5]. Physiological postpartum changes may disturb the process of fluid shift into systemic circulation because of increasing aortocaval compression and thus present a potential risk for prone positioning in patients post‐caesarean section with ARDS. This mechanism may have contributed to our patient's hypotension aetiologies. The increase of abdominal pressure while performing prone positioning could decrease internal organs' perfusion especially vessel‐rich organs such as gravid uterus, kidney, and bowel; however, none of the uterine atony‐related risk factors have been reported for prone positioning. In our patient, due to the high mortality risk from severe ARDS with uncontrolled bleeding condition, the veno‐venous extracorporeal membrane oxygenation (VV‐ECMO) was not considered as a preferred option. The prone positioning was applied after ventilator manoeuvre failure to rescue our patient's oxygenation. Significant improvement of ARDS was demonstrated after 20 h of procedure. As a result of infection and bleeding being controlled, the need for ventilator support was gradually decreased without re‐positioning and eventually the patient could be extubated. Our case illustrates a significant improvement from prone positioning for rescuing oxygenation and allowing the patient to undertake decompressive laparotomy successfully.

### Disclosure Statement

Appropriate written informed consent was obtained for publication of this case report and accompanying images.
